# Discriminating Active Tuberculosis from Latent Tuberculosis Infection by flow cytometric measurement of CD161-expressing T cells

**DOI:** 10.1038/srep17918

**Published:** 2015-12-08

**Authors:** Qianting Yang, Qian Xu, Qi Chen, Jin Li, Mingxia Zhang, Yi Cai, Haiying Liu, Yiping Zhou, Guofang Deng, Qunyi Deng, Boping Zhou, Hardy Kornfeld, Xinchun Chen

**Affiliations:** 1 Guangdong (Shenzhen) Key Laboratory for Diagnosis & Treatment of Emerging Infectious Diseases; 2 Shenzhen Key Laboratory of Infection & Immunity, Shenzhen Third People’s Hospital, Guangdong Medical College, China; 3 Institute of Microbiology, Chinese Academy of Sciences, China; 4 Institute of Pathogen Biology, Chinese Academy of Medical Sciences, China; 5 Department of Respiratory Diseases, Shenzhen Futian Hospital, China; 6 Department of Medicine, University of Massachusetts Medical School, USA

## Abstract

Interferon-gamma Release Assays (IGRAs) significantly increases the possibility for early diagnosis of tuberculosis, but IGRAs alone cannot discriminate active TB from LTBI. Therefore, fast and reliable discrimination of active tuberculosis, especially bacteriology negative tuberculosis, from LTBI is a great necessity. Here we established an assay based on flow cytometric multiparameter assay assessing expression of CD161 along with CD3, CD4, and CD8, whereby a set of indices formulated by the percentages of CD3^+^CD161^+^, CD3^+^CD4^+^CD161^+^ and CD3^+^CD8^+^CD161^+^ T cells multiplied with lymphocyte/monocyte ratio were established. Application of the CD3^+^CD8^+^CD161^+^ index to compare a cohort of active tuberculosis with a cohort of LTBI or health control yielded 0.7662 (95% confidence interval [CI] 0.6559–0.8552) or 0.7922 (95%  CI 0.6846–0.8763) for sensitivity and 0.9048 (95%  CI 0.8209–0.9580) or 0.8939 (95% CI 0.8392–0.9349) for specificity when the TB cohort was AFB^+^; the corresponding results were 0.7481 (95%  CI 0.6648–0.8198) or 0.7557 (95%  CI 0.6730–0.8265) for sensitivity and 0.8571 (95%  CI 0.7637–0.9239) or 0.8603 (95%  CI 0.8008–0.9075) for specificity when the TB cohort was AFB^−^. Our results reveal that in combination with IGRAs, CD161-based indices provide a novel, fast diagnostic solution addressing the limitation of current tuberculosis diagnostics.

Tuberculosis remains a severe global health threat. In 2013, there were 9 million incident cases and 1.5 million deaths^1^, approximately 95% of which occur in developing countries. Despite intensive research effort and tremendous progresses, rapid and accurate diagnosis of active tuberculosis remains difficult^1^. In recent years, enormous effort has been paid in applying techniques of nucleic acid^2^, proteomics^3^,^4^ and immunological biomarkers^5^ for diagnosis of tuberculosis. Among these, Xpert MTB/RIF (Cepheid, Sunnyvale, California), which is based on nucleic acid amplification, attracts considerable attention due to its fast and specific results for tuberculosis diagnosis and concomitant identification of rifampicin resistance, but its efficacy for those patients without sputum or AFB (acid fast bacilli) smear-negative was limited^4^,^6^,^7^,^8^. *Mycobacterium tuberculosis* (*Mtb*)-specific IFN-γ Release assays (IGRAs) by peripheral blood constitute an increasingly widely applied route^9^,^10^ and has been recognized as the most important advancement in immunodiagnosis of TB in the last decade. One major limitation with IGRAs, however, is that it cannot differentiate active tuberculosis from latent tuberculosis infection (LTBI)^9^,^10^,^11^. Attempts have been made to address this problem including integration of CD38^12^, CD27^13^ as well as CD4 and CD8 levels^14^ with the IFN-γ readout, but the diagnostic performance of these methods remains to be further validated by large cohorts.

Given the increasingly wide clinical adoption of IGRAs, we propose here an alternative solution to this diagnostic limitation, namely a conditional two-step strategy that first employs regular IGRAs to establish *Mtb* infection followed by a quick assay to differentiate active tuberculosis from LTBI. As IGRAs requires incubation with Esat-6 and/or CFP10 antigens for about one day to detect antigen-specific T cells responses, we hope to establish a fast and simple ensuing assay without such antigen-stimulation. With this in mind, we focused on evaluating the surface markers on T cells for their potential in TB diagnosis, since *Mtb* infection induces profound T cells responses, a mechanism on which most available TB immuno-diagnostic assays are based. Hence, a large panel of molecules including CD36, CD38, CD44, CD263, CD55, CD33, CD18, and CD161 was screened using flow cytometry; these markers were selected according to our previous mRNA microarray data^15^ as well as previous reports on the potential usefulness of some molecules^12^,^16^,^17^. Our preliminary data indicated that among the aforementioned molecules, CD161 was the best performing marker for discriminating active TB from LTBI.

Here we present the results of applying CD161 to discriminate active TB from LTBI, which indicate that the marker is promising for clinical application. Specifically, the percentages of CD161 expressing T cells including CD3^+^CD161^+^, CD3^+^CD4^+^CD161^+^ or CD3^+^CD8^+^CD161^+^ T cells were significantly lower in TB than in LTBI and healthy controls (HC). Nevertheless, the percentages of CD161-expressing T cells alone were not sufficient for effective differentiation of active TB from LTBI. To improve its performance, we incorporated the ratio of lymphocytes to monocytes, which is also decreased in patients with active TB^18^,^19^, to establish a set of multifactorial indices. Using two independent study groups we confirmed that the multifactorial indices have high performance in discriminating active TB from LTBI.

## Results

### The frequencies of CD161-expressing T cells were significantly decreased in patients with active TB

**Figure 1:**
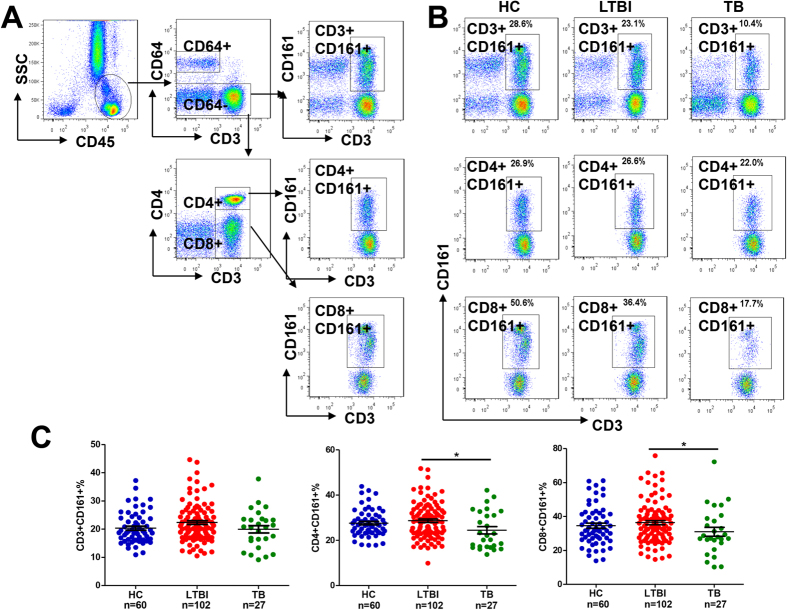
Establishment of a CD161-based immunological measure to diagnose active tuberculosis. (**A**) Gating strategy to enumerate CD161^+^ populations and the proposed formulas to compare active tuberculosis and LTBI or healthy control. Lymphocytes (CD45^+^CD64^−^) and monocytes (CD45^+^CD64^+^) were gated by the CD45 marker and then separated by the presence and absence of CD64. The different subsets of lymphocytes were gated via different combinations of CD3, CD4, and CD8 markers. Note CD4^+^CD161^+^ and CD8^+^CD161^+^ cells are also CD3^+^. (**B**) Gating and enumeration of CD161^+^ populations from specimens of 9 participants, consistently showing clear separation of cell groups. (**C**) Comparison between the LTBI or HC cohort and the TB cohort using the percentages of CD3^+^CD161^+^, CD4^+^CD161^+^ and CD8^+^CD161^+^ cells. *P < 0.05.

The FACS (fluorescence-activated cell sorting) gating strategy is illustrated in Fig. 1A. Lymphocytes (CD45^+^CD64^−^) and monocytes (CD45^+^CD64^+^) were gated based on the CD45 marker and the lower scale of side scatter and were subsequently differentiated by the CD64 marker (the large CD45^+^ population with the higher scale of side scatter being granulocytes). Afterwards, gating of CD3^+^CD161^+^, CD3^+^CD4^+^CD161^+^ and CD3^+^CD8^+^CD161^+^ lymphocytes was performed as illustrated in the workflow (Fig. 1A) and these distinct populations were consistently identified in blood samples from different individuals (Fig. 1B). Additionally, we demonstrated that the expression of CD161 on T cells was stable, as the percentages of CD161-expressing T cells showing no appreciable difference between the samples analyzed at different 24-hour intervals (Supplementary Figure 1). In comparison with HC and LTBI, patients with active tuberculosis had noticeably lower frequencies of CD161-expressing T cells. Nevertheless, these frequencies *per se* could not effectively differentiate active TB from LTBI/HC, as a high proportion of overlapping individuals were present between different groups (Fig. 1C).

### Establishment of indices of CD161-based T-cell response to detect active tuberculosis

**Figure 2:**
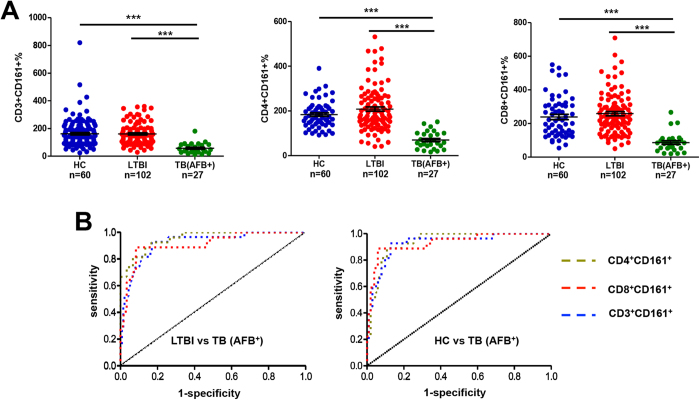
Comparison between the cohort of active tuberculosis and the cohort of LTBI or HC in study group I. (**A**) The analytic data of using each index to compare the cohorts of TB and HC or LTBI. (**B**) The ROC analysis of each index to distinguish TB from HC or LTBI. The three formulas below were used for examining the diagnostic performance for discriminating active tuberculosis from LTBI or HC. ***P < 0.0001.

In an attempt to investigate the clinical usefulness of CD161-expressing T cells for identifying active tuberculosis, we examined whether a score incorporating the ratio of lymphocytes to monocytes to the percentages of CD161-expressing T cells would generate an improved performance, since the ratio of lymphocytes to monocytes is also significantly decreased in patients with active TB^18^,^19^. Hence, we introduced a set of indices formulated by CD64^−^%/CD64^+^% (representing the ratio of lymphocytes to monocytes) multiplied with percentage of CD3^+^CD161^+^ cells, CD3^+^CD4^+^CD161^+^ cells or CD3^+^CD8^+^CD161^+^ cells. For simplicity, the latter two populations were referred as CD4^+^CD161^+^ and CD8^+^CD161^+^ hereafter. Application of the three indices in study group I, the training set, all convincingly differentiated the AFB^+^TB cohort from the cohort of HC or LTBI, as evidenced by the AUC (area under curve) values between 0.9130 (95% CI 0.8440–0.9819)–0.9543 (95% CI 0.9145–0.9941), sensitivity between 0.8148 (95% CI 0.6192–0.9370)–0.8889 (95% CI 0.7084–0.9765), specificity between 0.8997 (95% CI 0.8246–0.9369)–0.9412 (95% CI 0.8764–0.9781), and odds ratio between 7.39–15.11 (Fig. 2 and Supplementary Table 2). Although the three indices generated similar diagnostic performance, the CD8^+^CD161^+^ index overall displayed slightly better outcome than the CD3^+^CD161^+^ and CD4^+^CD161^+^ ones and was therefore the focus of the rest of the study.

### Validation of the CD161-based indices for Discriminating Active TB from LTBI by Independent Cohorts

**Figure 3:**
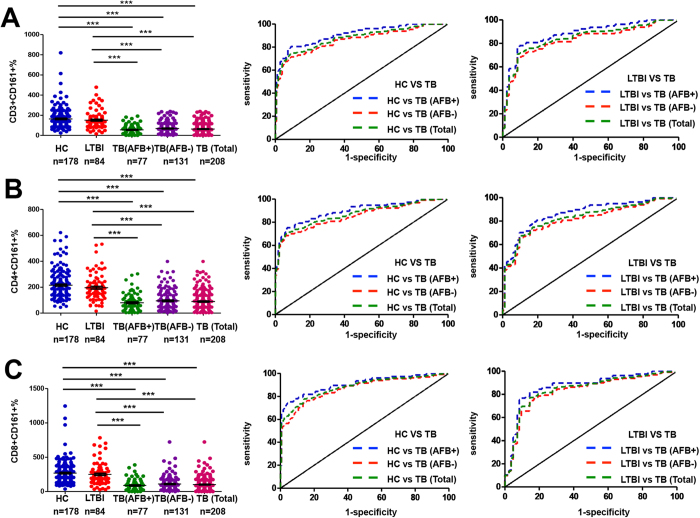
Comparison between the cohort of active tuberculosis and the cohort of LTBI or HC in study group II. (**A**–**C**) represent the results derived from the CD3^+^CD161^+^, CD4^+^CD161^+^ and CD8^+^CD161^+^ indices, respectively. For each panel, the left plot shows the analytic data of using each index to compare the different cohorts whereas the middle and right plots show the ROC analysis of each index to distinguish TB from HC or LTBI. ***P < 0.0001.

We then evaluated the diagnostic performance of the CD161-based indices more thoroughly by examining both AFB^+^TB and AFB^-^TB cohorts in the study group II. Not surprisingly, the indices consistently differentiated AFB^+^TB from LTBI or HC. For example, the CD8^+^CD161^+^ index generated the AUC = 0.8672 (95% CI 0.8079–0.9264) or 0.9073 (95% CI 0.8628–0.9519), sensitivity = 0.7662 (95% CI% CI 0.6559–0.8552) or 0.7922 (95% CI 0.6846–0.8763), specificity = 0.9048 (95% CI 0.8209–0.9580) or 0.8939 (95% CI 0.8392–0.9349), and odds ratio = 8.05 or 7.46 (Fig. 3 and Supplementary Table 3). Similar results were obtained when the CD3^+^CD161^+^ and CD4^+^CD161^+^ indices were employed (Fig. 3 and Supplementary Table 3).

Importantly, these indices also generated comparable performance for discriminating AFB^−^TB from LTBI or HC (AUC = 0.8304 [95% CI 0.7729–0.8879] or 0.8672 [95% CI 0.8077–0.8994] , sensitivity = 0.7481 [95% CI 0.6648–0.8198] or 0.7557 [95% CI 0.6730–0.8265], specificity = 0.8571 [95% CI 0.7637–0.9239] or 0.8603 [95% CI 0.8008–0.9075], and odds ratio = 5.24 or 5.41 for the CD8^+^CD161^+^ index) (Fig. 3 and Supplementary Table 3). When the AFB^+^TB and AFB^−^TB subjects were combined to compare with LTBI or HC, the results were in similar range (for the CD8^+^CD161^+^ index, AUC = 0.8444 [95% CI 0.7932–0.8949] or 0.8821[95% CI 0.8485–0.9157] , sensitivity = 0.7740 [95% CI 0.7111–0.8290] or 0.7115 [95% CI 0.6448–0.7721], specificity = 0.8571 [95% CI 0.7637–0.9239] or 0.9050 [95% CI 0.8523–0.9437], and odds ratio = 5.42 or 7.49).

### Clinical Application of the CD161-based indices in cohorts of active tuberculosis

**Figure 4:**
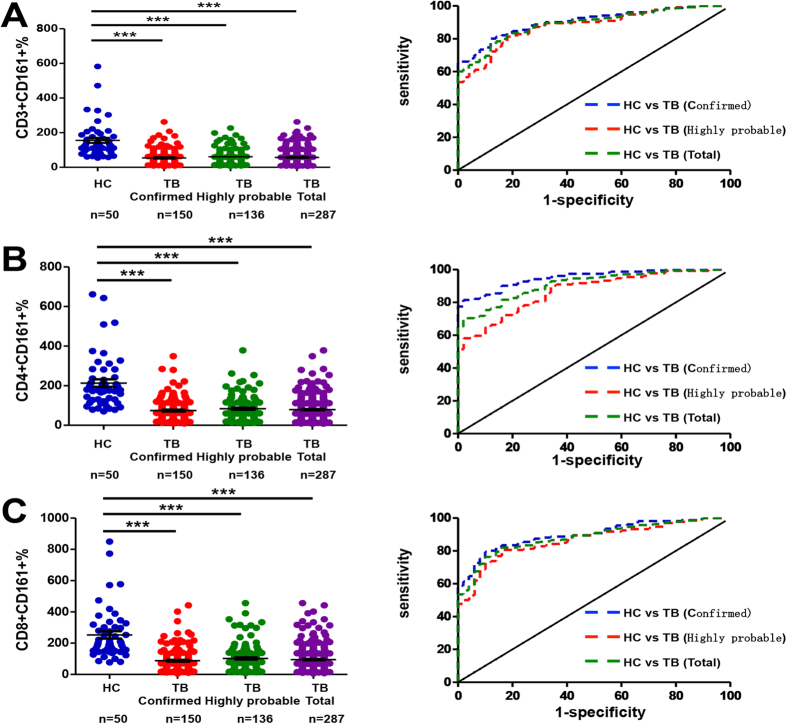
Comparison between the cohort of active tuberculosis and the cohort of HC in study group III. (**A–C**) represent the results derived from the CD3^+^CD161^+^, CD4^+^CD161^+^ and CD8^+^CD161^+^ indices, respectively. For each panel, the left plot shows the analytic data of using each index to compare the different cohorts whereas the right plot shows the ROC analysis of each index to distinguish TB from HC. ***P < 0.0001.

We next examined the performance of the three indices in a clinical setting-oriented study group III. For this purpose, each TB subject was categorized as confirmed tuberculosis if positive *Mtb* culture was obtained or highly probable tuberculosis if the specimen was culture-negative, but he/she met one of several active tuberculosis-indicative criteria (see Materials and Methods for details). Because HC and LTBI subjects behaved similarly with this method (Figs 2 and 3 and Supplementary Tables 2 and 3), only the former was recruited in this study group. Our analyses revealed that the index displayed excellent performance in discriminating healthy controls from TB cohorts (confirmed TB, highly probable TB and their combination) (Fig. 4 and Supplemental Table 4, AUC = 0.8969 [95% CI 0.8543–0.9396], 0.8661 [95% CI 0.8141–0.9181] or 0.8824 [95% CI 0.8425–0.9222], sensitivity = 0.7933 [95% CI 0.8726–0.9628], 0.7313 [95% CI 0.6480–0.8042] or 0.7641 [95% CI 0.7103–0.8122], specificity = 0.9 [95% CI 0.7819–0.9667], 0.9 [95% CI 0.7819–0.9667] or 0.9 [95% CI 0.7819–0.9667], and odds ratio = 7.93, 7.31 or 7.64, respectively, for the CD8^+^CD161^+^ index). Similar results were obtained when the CD3^+^CD161^+^ and CD4^+^CD161^+^  indices were employed (Fig. 4 and Supplementary Table 4).

Given the above results showing the efficacy of the CD161 for differentiating TB from LTBI, we performed a complete two-step procedure of IGRAs and CD161 assay for the study group IV. All 103 individuals were tested with the IFN-γ release assay and 65 cases of IGRAs positive samples were then examined for CD161-expressing T cells. To calculate the cut-off value of CD8^+^CD161^+^  index, an ROC curve was plotted using the LTBI and TB data of the study groups I and II, whereby the value was set to be 126.3% (<126.3% meaning active TB, Supplementary Table 5). Using this cut-off value, the ratio of positive of CD8^+^CD161^+^  index was 15.38% (2/13) for LTBI, 75%(33/44) for TB and 0(0/1) for non-TB pneumonia (bacterial pneumonia). Hence, our results demonstrated that the CD161 method can reliably identify active TB subjects once *Mtb* infection is established by IGRAs examination.

## Discussion

Before effective vaccines against TB become available, early diagnosis of active TB followed by short-term anti-TB treatment will remain a major strategy for TB control. Correspondingly, great effort has been dedicated to seeking sensitive, fast, and reliable TB diagnostic tools. Two methods, namely Xpert MTB/RIF for detection of the pathogen, and IGRAs for immune-diagnosis of its infection, have been developed as the most important achievements over decades and are increasingly applied in recent years^20^. Although Xpert MTB/RIF shows significant enhancement in sensitivity compared to conventional methods, it requires sputum that is unavailable in some proportion of patients and produces suboptimal results for AFB^−^TB patients^4^,^6^,^7^,^8^. On the other hand, IGRAs have no issues in sample preparation but cannot distinguish active TB from LTBI. This is a major defect that limits its clinical application, because the treatments for active TB are considerably different from those of LTBI. For example, in many countries with high TB burden such as China, LTBI is typically not subjected to preventive anti-TB chemotherapy^9^,^10^,^11^. To overcome this limitation, several additional markers have been explored to work in combination with IFN-γ and some have produced considerable diagnostic improvements^12^,^13^,^14^. As an alternative solution, we report here that a fast CD161-expressing T cells-based indices was useful to discriminate active TB from LTBI, which generate high sensitivity and specificity.

Besides CD4 and CD8^14^,^21^, the immunological markers implicated in identification of active tuberculosis also include CD38, CD26, CD27, CD64 and CD161^12^,^13^,^17^,^22^. Except a very recently published study on CD38^12^, no cohort study with statistically sufficient sample sizes has been dedicated to examining the diagnostic performance of these markers (e.g., the CD27 study^17^ having a group of 21 TB contacts, including only 10 being IFN-γ positive). CD161 is a C-type lectin-like receptor expressed by a broad range of lymphocytes, including CD4^+^, CD8^+^, γδ^+^ T-cells, NK cells, and mucosal-associated invariant T (MAIT) cells. Consistent with the extraordinary heterogeneity of CD161-expressing cells, the biological functions of the marker including its roles in mycobacterial infections are not well understood. Nevertheless, the decrease of CD161-expressing CD4 T cells in active TB patients was in line with our previous results that the suppressed Th17 responses were associated with the disease^23^. In consistence, it was reported that almost all Th17 cells were CD161-positive^24^,^25^. Another important group of CD161-expressing cells is MAIT, which has been clinically shown to play crucial roles in patients with severe bacterial infections^26^. MAIT cells represent the most abundant innate-like T-cell population within human body, comprising up to ~5% of the total T-cell population and are involved in anti-bacterial immunity. Recently, it was shown that patients with active tuberculosis had significantly lower percentage of CD161-expressing CD8^+^TRAV1–2^+^ MAIT cells than those of uninfected and LTBI subjects^16^. This finding appeared consistent with our data, but it enlisted miniscule sample sizes (6 uninfected, 3 TB and 3 LTBI). In comparison, much greater sample scale was recruited in this study, thus our evidence regarding the lower CD161 expression level in active tuberculosis subjects was much more convincing. Collectively, our results and other evidence suggested that CD161-expressing lymphocytes are important for protective immunity in preventing the progression of active TB from *Mtb* infection. Further investigations are warranted to clarify its roles as well as the underlying mechanisms.

Of note, the CD161-based immunological assay established herein has several features suitable for clinical application. First, the three CD161-based indices displayed reliable capacity in discriminating active tuberculosis from latent infection. Second, the blood samples collected from participants can be kept at room temperature for 24 h and this duration does not affect the assay outcome (supplementary Figure 1). Third, the cell groups during the FACS process are well separated and there is no gating ambiguity (Fig. 1A,B), which is own to the distinguishable CD161 expression on the surface of T cells. Fourth, the assay established in this lab, is cost-effective and has a price tag of 30 RMB (~4.8 USD) per test. Lastly, the performance of this assay is comparable for both AFB^−^ and AFB^+^ TB subjects. Nonetheless, we are aware that there are some limitations regarding to its clinical application, a major part of which is due to the fact that flow cytometry devices are not readily available in common hospitals worldwide, especially in developing countries like China.

## Methods

### Study Groups

We enlisted three study groups (Supplementary Table 1) to investigate three diagnostic indices integrating CD161 response with that of CD3, CD4 or CD8. The general information and clinical characteristics of the recruited participants are summarized in Supplementary Table 1. The study was approved by the Institutional Review Board of Shenzhen Third People’s Hospital and the methods were carried out in accordance with the approved guidelines of the institution. Written informed consent was obtained from all participants.

For the study groups I, II, and IV, the diagnosis of the active tuberculosis participants was based on laboratory isolation of *Mtb* on smear of acid fast bacilli (AFB), and/or *Mtb* culture from sputum, and/or enzyme-linked immunospot assay (ELISpot), and/or radiological images of classical tuberculosis, and/or histological evidence of tuberculosis, and/or PCR positive for *Mtb*. In addition, a pneumonia subject was defined as a patient with pulmonary inflammation but lacking any microbiological or histological evidence of TB, or confirmation of an alternative diagnosis. All LTBI subjects, who were family members of tuberculosis patients, were asymptomatic and had T-cell responses specific to IFN-γ detected by ELISpot, as described previously^27^. For the study group III, the confirmed tuberculosis subjects had positive sputum culture of *Mtb*, whereas the cases of highly probable tuberculosis were culture negative but met one of the following criteria: radiological images of classical tuberculosis, AFB smear positive, histological evidence of tuberculosis, or PCR positive for *Mtb*.

### Flow Cytometry Analyses

Fresh whole blood (200 μl) was collected from participants of HC, LTBI, and TB. Each blood specimen was associated with a unique patient identification number, which did not reveal the sample types (i.e., HC, LTBI, and TB). The investigators performing flow cytometry were blinded to the clinical diagnoses. Red blood cells were lysed with lysis solution (BD Bioscience). The samples were stained with surface mAbs against CD3 (BD Bioscience, SY7), CD4 (BD Bioscience, SY3), CD45 (BD Bioscience, 2D1), CD161 (Beckman Coulter, 191B8), and CD64 (Beckman Coulter, 22). After incubating for 15 min at room temperature in the dark, the samples were rinsed and prepared for analysis. Samples were acquired on BD FACSCanto II flow cytometry system (BD) and data were analyzed using FlowJo software.

### Formulas of the indices

The three indices were calculated according to the following formulas: Index I = (CD64^−^%/CD64^+^ %) X CD3 ^+^ CD161^+^ %; Index II  = (CD64^−^%/CD64 ^+^ %) X CD3^+^ CD4^+^ CD161^+^ %; Index III = (CD64^−^%/CD64^+^%) X CD3^+^CD8^+^CD161^+^%.

### Statistical Analyses

All statistical tests were performed with Prism 5.0 (GraphPad). Unpaired t-test was used to analyze the difference between two groups. Differences were considered significant if P < 0.05. Receiver operating characteristic (ROC) analysis was performed to determine the power of each indices to distinguish TB from HC and TB from LTBI. The ROC curves were constructed by plotting the true positive samples (sensitivity) against the false-positive samples (1-specificity) for each possible cutoff point. Areas under the curve (AUC) were calculated along with their 95% confidence intervals (95% CI) by using a nonparametric approach. Cutoff value of CD8 ^+^ CD161^+^  index was estimated at various sensitivities and specificities. To avoid false-positive results, a cutoff value corresponding to the maximum Youden index, defined as sensitivity+specificity-1, was retained. Optimal cutoff levels was determined by ROC curve analysis, based on the highest likelihood ratio^28^.

## Additional Information

**How to cite this article**: Yang, Q. *et al.* Discriminating Active Tuberculosis from Latent Tuberculosis Infection by flow cytometric measurement of CD161-expressing T cells. *Sci. Rep.* **5**, 17918; doi: 10.1038/srep17918 (2015).

## Supplementary Material

Supplementary Information

## References

[b1] WHO. Global tuberculosis control: WHO (World Health Organization) report 2013. (World Health Organization, 2013).

[b2] Moure R. *et al.* Rapid detection of *Mycobacterium tuberculosis* complex and rifampin resistance in smear-negative clinical samples by use of an integrated real-time PCR method. J Clin Microbiol 49, 1137–1139 (2011). 21191053 10.1128/JCM.01831-10 PMC3067676

[b3] Berry M. P. *et al.* An interferon-inducible neutrophil-driven blood transcriptional signature in human tuberculosis. Nature 466, 973–977 (2010). 20725040 10.1038/nature09247 PMC3492754

[b4] Zeka A. N., Tasbakan S. & Cavusoglu C. Evaluation of the GeneXpert MTB/RIF assay for rapid diagnosis of tuberculosis and detection of rifampin resistance in pulmonary and extrapulmonary specimens. J Clin Microbiol 49, 4138–4141 (2011). 21956978 10.1128/JCM.05434-11 PMC3232962

[b5] Harari A. *et al.* Dominant TNF-alpha+ *Mycobacterium tuberculosis*-specific CD4+ T cell responses discriminate between latent infection and active disease. Nat Med 17, 372–376 (2011). 21336285 10.1038/nm.2299 PMC6570988

[b6] Armand S., Vanhuls P., Delcroix G., Courcol R. & Lemaître N. Comparison of the Xpert MTB/RIF Test with an IS6110-TaqMan real-time PCR assay for direct detection of *Mycobacterium tuberculosis* in respiratory and nonrespiratory specimens. J Clin Microbiol 49, 1772–1776 (2011). 21411592 10.1128/JCM.02157-10 PMC3122654

[b7] Kokuto H. *et al.* Detection of *Mycobacterium tuberculosis* (MTB) in fecal specimens from adults diagnosed with pulmonary tuberculosis using the Xpert MTB/rifampicin test. *Open Forum* Infect Dis 2, ofv074, 10.1093/ofid/ofv074 (2015). PMC4462888 26125035

[b8] Kim M. J., Nam Y. S., Cho S. Y., Park T. S. & Lee H. J. Comparison of the Xpert MTB/RIF assay and real-time PCR for the detection of *Mycobacterium tuberculosis*. Ann Clin Lab Sci 45, 327–332 (2015). 26116598

[b9] Herrera V., Perry S., Parsonnet J. & Banaei N. Clinical application and limitations of interferon-γ release assays for the diagnosis of latent tuberculosis infection. Clin Infect Dis 52, 1031–1037 (2011). 21460320 10.1093/cid/cir068

[b10] Meier T., Eulenbruch H. P., Wrighton-Smith P., Enders G. & Regnath T. Sensitivity of a new commercial enzyme-linked immunospot assay (T SPOT-TB) for diagnosis of tuberculosis in clinical practice. Eur J Clin Microbiol Infect Dis 24, 529–536 (2005). 16133410 10.1007/s10096-005-1377-8

[b11] Jasmer R. M., Nahid P. & Hopewell P. C. Latent Tuberculosis Infection. N Eng J Med 347, 1860–1866 (2002). 10.1056/NEJMcp021045 12466511

[b12] Adekambi T. *et al.* Biomarkers on patient T cells diagnose active tuberculosis and monitor treatment response. J Clin Invest 125, 1827–1838 (2015). 25822019 10.1172/JCI77990 PMC4598074

[b13] Portevin D. *et al.* Assessment of the novel T-cell activation marker-tuberculosis assay for diagnosis of active tuberculosis in children: a prospective proof-of-concept study. Lancet Infect Dis 14, 931–938 (2014). 25185458 10.1016/S1473-3099(14)70884-9

[b14] Rozot V. *et al.* Combined use of *Mycobacterium tuberculosis*-specific CD4 and CD8 T-cell responses is a powerful diagnostic tool of active tuberculosis. Clin Infect Dis 60, 432–437 (2015). 25362202 10.1093/cid/ciu795 PMC4293395

[b15] Cai Y. *et al.* Increased complement C1q level marks active disease in human tuberculosis. PLoS ONE 9, e92340, 10.1371/journal.pone.0092340 (2014). 24647646 PMC3960215

[b16] Sharma P. K. *et al.* High expression of CD26 accurately identifies human bacteria-reactive MR1-restricted MAIT cells. Immunology, 10.1111/imm.12461 (2015). PMC4479542 25752900

[b17] Nikitina I. Y. *et al.* *Mtb*-Specific CD27^low^ CD4 T cells as markers of lung tissue destruction during pulmonary tuberculosis in humans. PLoS ONE 7, e43733, 10.1371/journal.pone.0043733 (2012). 22937086 PMC3427145

[b18] Naranbhai V. *et al.* Ratio of monocytes to lymphocytes in peripheral blood identifies adults at risk of incident tuberculosis among HIV-infected adults initiating antiretroviral therapy. J Infect Dis 209, 500–509 (2014). 24041796 10.1093/infdis/jit494 PMC3903371

[b19] Naranbhai V. *et al.* The association between the ratio of monocytes:lymphocytes at age 3months and risk of tuberculosis (TB) in the first two years of life. BMC Medicine 12, 120 (2014). 25034889 10.1186/s12916-014-0120-7 PMC4223414

[b20] Pai M. & Schito M. Tuberculosis diagnostics in 2015: landscape, priorities, needs, and prospects. J Infect Dis 211, S21–S28 (2015). 25765103 10.1093/infdis/jiu803 PMC4366576

[b21] Rozot V. *et al.* *Mycobacterium tuberculosis*-specific CD8+ T cells are functionally and phenotypically different between latent infection and active disease. Eur J Immunol 43, 1568–1577 (2013). 23456989 10.1002/eji.201243262 PMC6535091

[b22] Sutherland J. S. *et al.* Differential gene expression of activating Fcγ receptor classifies active tuberculosis regardless of human immunodeficiency virus status or ethnicity. Clin Microbiol Infect 20, O230–O238 (2014). 24205913 10.1111/1469-0691.12383

[b23] Chen X. *et al.* Reduced Th17 response in patients with tuberculosis correlates with IL-6R expression on CD4+ T Cells. Am J Respir Crit Care Med 181, 734–742 (2010). 20019339 10.1164/rccm.200909-1463OC

[b24] Guedan S. *et al.* ICOS-based chimeric antigen receptors program bipolar TH17/TH1 cells. Blood 124, 1070–1080 (2014). 24986688 10.1182/blood-2013-10-535245 PMC4133482

[b25] Cosmi L. *et al.* Human interleukin 17–producing cells originate from a CD161^+^CD4^+^ T cell precursor. J Exp Med 205, 1903–1916 (2008). 18663128 10.1084/jem.20080397 PMC2525581

[b26] Grimaldi D. *et al.* Specific MAIT cell behaviour among innate-like T lymphocytes in critically ill patients with severe infections. Intensive Care Med 40, 192–201 (2014). 24322275 10.1007/s00134-013-3163-x

[b27] Zhang M. *et al.* Diagnosis of latent tuberculosis infection in bacille Calmette-Guerin vaccinated subjects in China by interferon-gamma ELISpot assay. Int J Tuberc Lung Dis 14, 1556–1563 (2010). 21144240

[b28] Yang Q. *et al.* IP-10 and MIG are compartmentalized at the site of disease during pleural and meningeal tuberculosis and are decreased after antituberculosis treatment. Clin Vaccine Immunol 21, 1635–1644 (2014). 25274803 10.1128/CVI.00499-14 PMC4248780

