# Supplementary material for: Discriminating Active Tuberculosis from Latent Tuberculosis Infection by flow cytometric measurement of CD161-expressing T cells
Source: Sci Rep. 2015 Dec 8;5:17918. doi: 10.1038/srep17918 (PMC4672319; doi:10.1038/srep17918)
Supplement: Supplementary Information [file srep17918-s1.doc]

**Discriminating Active Tuberculosis from Latent Tuberculosis Infection by flow cytometric measurement of CD161-expressing T cells**

Qianting Yang, Qian Xu, Qi Chen, Jin Li, Mingxia Zhang, Yi Cai, Haiying Liu, Yiping Zhou, Guofang Deng, Qunyi Deng, Boping Zhou, Hardy Kornfeld and Xinchun Chen

**Table S1. General information of the participants.**

| **Study Group** | **Subject** | **Case No.** | **Sex (M/F)** | **Age (Years)** | **Cases of sputum culture tested/positive (ratio)** | | **AFB cases tested/ positive (ratio)** | **Elispot cases tested/positive (ratio)** |
| --- | --- | --- | --- | --- | --- | --- | --- | --- |
| Ⅰ | HC  LTBI  TB | 60  102  27 | 44/16  82/20  17/10 | 28.46±0.84  35.06±0.75  39.96±2.74 | NA  NA  27/9 (33.33%) | NA  NA  27/27 (100%) | | 60/0 (0)  102/102 (100%)  7/6 (85.71%) |
| Ⅱ | HC  LTBI  TB | 178  84  208 | 94/84  62/22  145/63 | 35.06±0.75  31.32±0.99  36.58±1.03 | NA  NA  178/47 (26.40%) | NA  NA  208/77 (37.02%) | | 178/0 (0)  84/84 (100%)  144/118 (81.94%) |
| Ⅲ | HC  Highly Prob. TB  Confirmed TB | 50  136  150 | 28/22  92/44  91/59 | 34.24±1.55  38.74±1.62  40.53±1.42 | NA  8/0 (0)  150/150 (100%) | NA  44/6 (13.64%)  148/69 (48.25%) | | 50/0 (0)  103/75 (72.82%)  127/100 (78.74%) |
| Ⅳ | HC or LTBI  TB  Pneumonia | 43  52  8 | 12/31  40/12  5/3 | 43.23±1.79  40.83±2.42  49±5.39 | NA  30/7(23.33%)  8/0(0) | NA  32/8(25%)  8/0(0) | | 43/13（30.23%)  52/44（84.46%）  8/1(12.5%) |

**Table S2. Analytic results of using the CD161-based indices to compare TB and LTBI or HC cohorts in Group Ⅰ.**

| **Index** | **AUC**  **95% CI** | **P value** | **Sensitivity**  **95% CI** | **Specificity**  **95% CI** | **Odds ratio** |
| --- | --- | --- | --- | --- | --- |
|  |  | **LTBI vs AFB^+^TB** | |  |  |
| CD4^+^CD161^+^ | 0.9386  0.8982 - 0.9791 | <0.0001 | 0.8148  0.6192 - 0.9370 | 0.9216  0.8513 - 0.9655 | 10.39 |
| CD8^+^CD161^+^ | 0.9328  0.8770 - 0.9886 | <0.0001 | 0.8889  0.7084 - 0.9765 | 0.9412  0.8764 - 0.9781 | 15.11 |
| CD3^+^CD161^+^ | 0.9277  0.8712 - 0.9843 | <0.0001 | 0.8148  0.6192 - 0.9370 | 0.902  0.8271 - 0.9520 | 8.31 |
|  |  | **HC vs AFB^+^TB** | |  |  |
| CD4^+^CD161^+^ | 0.9543  0.9145 - 0.9941 | <0.0001 | 0.8148  0.6192 - 0.9370 | 0.9333  0.8380 - 0.9815 | 12.22 |
| CD8^+^CD161^+^ | 0.9130  0.8440 - 0.9819 | <0.0001 | 0.8889  0.7084 - 0.9765 | 0.9167  0.8161 - 0.9724 | 10.67 |
| CD3^+^CD161^+^ | 0.921  0.8653 - 0.9768 | <0.0001 | 0.8148  0.6192 - 0.9370 | 0.8897  0.8246 - 0.9369 | 7.39 |

**Table S3. Analytic results of using the CD161^+^-based indices to compare TB and LTBI or HC cohorts in Group Ⅱ.**

| **Comparison** | **AUC**  **95% CI** | **P value** | **Sensitivity**  **95% CI** | **Specificity**  **95% CI** | **Odds ratio** |
| --- | --- | --- | --- | --- | --- |
|  |  | **CD3^+^CD161^+^** | |  |  |
| LTBI vs TB (AFB^+^) | 0.8811  0.8273 - 0.9349 | <0.0001 | 0.7792  0.6702 - 0.8658 | 0.9048  0.8209 - 0.9580 | 8.18 |
| HC vs TB (AFB^+^) | 0.9081  0.8662 - 0.9501 | <0.0001 | 0.8052  0.6991 - 0.8867 | 0.9106  0.8589 - 0.9480 | 9.01 |
| LTBI vs TB (AFB^-^) | 0.8245  0.7682 - 0.8809 | <0.0001 | 0.7252  0.6404 - 0.7995 | 0.8571  0.7637 - 0.9239 | 5.08 |
| HC vs TB (AFB^-^) | 0.8536  0.8164 - 0.9006 | <0.0001 | 0.7099  0.6242 - 0.7859 | 0.9106  0.8589 - 0.9480 | 7.94 |
| LTBI vs TB (total) | 0.8455  0.7984 - 0.8925 | <0.0001 | 0.7163  0.6499 - 0.7765 | 0.9048  0.8209 - 0.9580 | 7.52 |
| HC vs TB (total) | 0.8738  0.8387 - 0.9088 | <0.0001 | 0.7452  0.6803 - 0.8029 | 0.9106  0.8589 - 0.9480 | 8.34 |
|  |  | **CD4^+^CD161^+^** | |  |  |
| LTBI vs TB (AFB^+^) | 0.8652  0.8081 - 0.9223 | <0.0001 | 0.7143  0.6000 - 0.8115 | 0.8690  0.7778 - 0.9328 | 5.45 |
| HC vs TB (AFB^+^) | 0.8957  0.8490 - 0.9423 | <0.0001 | 0.7532  0.6418 - 0.8444 | 0.9330  0.8858 - 0.9649 | 11.24 |
| LTBI vs TB (AFB^-^) | 0.8102  0.7524 - 0.8679 | <0.0001 | 0.7099  0.6242 - 0.7859 | 0.8452  0.7499 - 0.9149 | 4.59 |
| HC vs TB (AFB^-^) | 0.8452  0.7981 - 0.8924 | <0.0001 | 0.7099  0.6242 - 0.7859 | 0.8827  0.8263 - 0.9259 | 6.05 |
| LTBI vs TB (total) | 0.8305  0.7820 - 0.8791 | <0.0001 | 0.7308  0.6651 - 0.7898 | 0.8452  0.7499 - 0.9149 | 4.72 |
| HC vs TB (total) | 0.8821  0.8485 - 0.9157 | <0.0001 | 0.7115  0.6448 - 0.7721 | 0.9050  0.8523 - 0.9437 | 7.49 |
|  |  | **CD8^+^CD161^+^** | |  |  |
| LTBI vs TB (AFB^+^) | 0.8672  0.8079 - 0.9264 | <0.0001 | 0.7662  0.6559 - 0.8552 | 0.9048  0.8209 - 0.9580 | 8.05 |
| HC vs TB (AFB^+^) | 0.9073  0.8628 - 0.9519 | <0.0001 | 0.7922  0.6846 - 0.8763 | 0.8939  0.8392 - 0.9349 | 7.46 |
| LTBI vs TB (AFB^-^) | 0.8304  0.7729 - 0.8879 | <0.0001 | 0.7481  0.6648 - 0.8198 | 0.8571  0.7637 - 0.9239 | 5.24 |
| HC vs TB (AFB^-^) | 0.8672  0.8077 - 0.8994 | <0.0001 | 0.7557  0.6730 - 0.8265 | 0.8603  0.8008 - 0.9075 | 5.41 |
| LTBI vs TB (total) | 0.844  0.7932 - 0.8949 | <0.0001 | 0.7740  0.7111 - 0.8290 | 0.8571  0.7637 - 0.9239 | 5.42 |
| HC vs TB (total) | 0.8821  0.8485 - 0.9157 | <0.0001 | 0.7115  0.6448 - 0.7721 | 0.905  0.8523 - 0.9437 | 7.49 |

**Table S4. Analytic results of using the CD161^+^ -based indices to compare TB and HC cohorts in Group Ⅲ.**

| **Comparison** | **AUC**  **95% CI** | **P value** | **Sensitivity**  **95% CI** | **Specificity**  **95% CI** | | **Odds ratio** |
| --- | --- | --- | --- | --- | --- | --- |
|  |  | **CD3^+^CD16** |  | |  | |
| HC vs TB (confirmed) | 0.9044  0.8639 - 0.9449 | <0.0001 | 0.7467  0.6693-0.8141 | 0.9000  0.7819- 0.9667 | | 7.47 |
| HC vs TB (highly prob.) | 0.8797  0.8306 - 0.9288 | <0.0001 | 0.7537  0.6719 - 0.8240 | 0.86  0.7326 - 0.9418 | | 5.38 |
| HC vs TB (total) | 0.8927  0.8548-0.9307 | <0.0001 | 0.7852  0.7328-0.8315 | 0.86  0.7326 - 0.9418 | | 5.61 |
|  |  | **CD4^+^CD161^+^** | | | |  |
| HC vs TB (confirmed) | 0.9017  0.8596 - 0.9439 | <0.0001 | 0.7133  0.6339-0.7841 | 0.9  0.7819 - 0.9667 | | 7.13 |
| HC vs TB (highly prob.) | 0.8704  0.8190 - 0.9219 | <0.0001 | 0.6493  0.5621 - 0.7296 | 0.9  0.7819 - 0.9667 | | 6.49 |
| HC vs TB (total) | 0.8870  0.8461- 0.9278 | <0.0001 | 0.7148  0.6585- 0.7666 | 0.86  0.7326-0.9418 | | 5.11 |
|  |  | **CD8^+^CD161^+^** | | | |  |
| HC vs TB (confirmed) | 0.8969  0.8543 -.9396 | <0.0001 | 0.7933  0.8726 - 0.9628 | 0.9  0.7819 - 0.9667 | | 7.93 |
| HC vs TB (highly prob.) | 0.8661  0.8141 - 0.9181 | <0.0001 | 0.7313  0.6480 - 0.8042 | 0.9  0.7819 - 0.9667 | | 7.31 |
| HC vs TB (total) | 0.8824  0.8425-0.9222 | <0.0001 | 0.7641  0.7103-0.8122 | 0.9  0.7819 - 0.9667 | | 7.64 |

**Table S5. Analytic results of using the CD8^+^CD161^+^ index to compare TB and LTBI cohorts from groups Ⅰ and Ⅱ.**

| **Comparison** | **AUC**  **95% CI** | **P value** | **Cutoff value** | **Sensitivity**  **95% CI** | **Specificity**  **95% CI** | **Odds ratio** |
| --- | --- | --- | --- | --- | --- | --- |
| LTBI vs TB | 0.8702  0.8353-0.9051 | < 0.0001 | < 126.3% | 0.7532  0.6929-0.8069 | 0.871  0.8141-0.9155 | 5.84 |

**Supplementary Figure 1**

**Supplementary Figure 1. Comparison the percentages of CD161-expressing T cells between different time points.**

The CD161-expressing T cells of a same sample were detected in two time point 0h and 24h (keeping at room temperature for 24 h). And the analytic data of three indices were compared (n=17).
